# Supplementary material for: The Effect of Endometriosis on In Vitro Fertilization Outcomes: A Systematic Review and Meta-Analysis
Source: Healthcare (Basel). 2024 Dec 3;12(23):2435. doi: 10.3390/healthcare12232435 (PMC11641477; doi:10.3390/healthcare12232435)
Supplement: Supplementary file 1 [file healthcare-12-02435-s001.zip › healthcare-3264236-supplementary.pdf]

Supplementary material 1 List of the excluded studies and reasons for exclusion

Author	Year	Title	Reason for the exclusion
Viganò	2023	Conventional IVF performs similarly in women with and without endometriosis	Surgical intervention
Zimmermann	2023	Impact of moderate-to-severe endometriosis on IVF cumulative live birth rate: a retrospective matched cohort study	Surgical intervention
Li	2023	Pretreatment of Dienogest for Women with Endometriosis in in vitro Fertilization: A Systematic Review and Meta-Analysis	Sistematic review
Maignien	2023	Oocyte donation outcomes in endometriosis patients with multiple IVF failures	Oocyte donation
Gremillet	2023	Endometriosis, infertility and occupational life: women's plea for recognition	Not relevant outcomes
Gervoise-Boyer	2023	Risk factors associated with preterm birth in singletons born after IVF: a national cohort study	Not relevant outcomes
Tian	2023	Efficacy of long-term pituitary down-regulation pretreatment prior to in vitro fertilization in infertile patients with endometriosis: A meta-analysis	Sistematic review + not relevant outcomes
Zhu	2023	Development and validation of a nomogram model for predicting clinical pregnancy in endometriosis patients undergoing fresh embryo transfer	No control group
Paik	2023	Cumulative pregnancy rate via multiple fresh or frozen embryo transfers in women with current, resected, or recurred endometrioma	No control groups
Wenjing	2023	Xiaoyi Yusi decoction improves fertilization and embryo transfer outcomes in patients with endometriosis	Not relevant outcomes
Zareii	2023	Evaluation of endometrioma size effect on ovarian reserve, embryo quality and pregnancy outcome after in vitro fertilization cycle; a cross-sectional study	Not relevant outcomes
Yang	2023	Retrospective analysis of the endometrial preparation protocols for frozen-thawed embryo transfer cycles in women with endometriosis	Medical treatment
Dongye	2023	The Impact of Endometrioma on Embryo Quality in In Vitro Fertilization: A Retrospective Cohort Study	Surgical intervention
Li	2023	Analysis of cumulative live birth rate outcomes of three ovarian stimulation protocols in patients after laparoscopic cystectomy of ovarial endometrioma: a retrospective cohort study	Surgical intervention
Shao	2023	Impact of dienogest pretreatment on IVF-ET outcomes in patients with endometriosis: a systematic review and meta-analysis	Medical treatment
Esmaeilzadeh	2022	Stages of endometriosis: Does it affect oocyte quality, embryo development and fertilization rate?	Not available data
Lin	2022	Effect of atosiban on in vitro fertilization pregnancy outcome among women with endometriosis in presence or absence of adenomyosis	Medical treatment
Qu	2022	The effect of endometriosis on IVF/ICSI and perinatal outcome: A systematic review and meta-analysis	Sistematic review
Frangez	2022	Reproductive outcomes after laparoscopic surgery in infertile women affected by ovarian endometriomas, with or without *in vitro* fertilisation: results from the SAFE (surgery and ART for endometriomas) trial	Surgical intervention

Rees	2022	Women with combined adenomyosis and endometriosis on MRI have worse IVF/ICSI outcomes compared to adenomyosis and endometriosis alone: A matched retrospective cohort study	Surgical intervention and medical treatment
Ribot	2022	Pregnancy outcomes after in vitro fertilization for moderate and severe endometriosis. A case-control study	Not relevant outcomes:
Gao	2022	Effects of ovarian endometrioma aspiration on in vitro fertilization-intracytoplasmic sperm injection and embryo transfer outcomes: a systematic review and meta-analysis	Sistematic review
Bourdon	2022	Presence of adenomyosis at MRI reduces live birth rates in ART cycles for endometriosis	No control group
Arshad	2022	Comparative analysis of IVF-ICSI outcomes between advanced and early stage of endometriosis stimulated with HMG	No control group
Zhang	2021	Impact of Uterine Adenomyosis on Pregnancy Outcomes in Women Undergoing In Vitro Fertilization Treated With a Long-Term Pituitary Downregulation Protocol	No control group
Ferrier	2021	Use of the EFI score in endometriosis-associated infertility: A cost-effectiveness study	Surgical intervention
Casals	2021	Impact of Surgery for Deep Infiltrative Endometriosis before In Vitro Fertilization: A Systematic Review and Meta-analysis	Sistematic review + Surgical intervention
Alshehre	2021	The impact of endometrioma on in vitro fertilisation/intra-cytoplasmic injection IVF/ICSI reproductive outcomes: a systematic review and meta-analysis	Sistematic review
Robin	2021	Impact of endometriosis on oocyte morphology in IVF-ICSI: retrospective study of a cohort of more than 6000 mature oocytes	Surgical treatment
Muzii	2021	Endometriosis-associated infertility: surgery or IVF?	Sistematic review
Zhou	2020	Acupuncture for secondary dysmenorrhea of adenomyosis: a prospective case-series study	Not relevant outcomes
Boucret	2020	Endometriosis Lowers the Cumulative Live Birth Rates in IVF by Decreasing the Number of Embryos but Not Their Quality	Surgical intervention
Yland	2020	Endometrioma, the follicular fluid inflammatory network and its association with oocyte and embryo characteristics	Not relevant outcomes
Neal	2020	Three-dimensional ultrasound diagnosis of adenomyosis is not associated with adverse pregnancy outcome following single thawed euploid blastocyst transfer: prospective cohort study	Adenomyosis
Li	2020	Analysis of IVF/ICSI-FET Outcomes in Women With Advanced Endometriosis: Influence on Ovarian Response and Oocyte Competence	Surgical intervention
Yevseyevich Radzinsky	2019	Implantation failures in women with infertility associated endometriosis	No data available
Yang	2019	Risk of miscarriage in women with endometriosis undergoing IVF fresh cycles: a retrospective cohort study	Surgical intervention
Horton	2019	Reproductive, obstetric, and perinatal outcomes of women with adenomyosis and endometriosis: a systematic review and meta-analysis	Sistematic review
Sharma	2019	Pregnancy and Live Birth Rates Are Comparable in Young Infertile Women Presenting with Severe Endometriosis and Tubal Infertility	Surgical intervention
Orazov	2019	Oocyte quality in women with infertility associated endometriosis	Not relevant outcomes

Murta	2018	Endometriosis does not affect live birth rates of patients submitted to assisted reproduction techniques: analysis of the Latin American Network Registry database from 1995 to 2011	No data available
Coccia	2018	Bilateral Endometrioma Excision: Surgery- Related Damage to Ovarian Reserve	Surgical intervention
Bourdon	2018	The deferred embryo transfer strategy improves cumulative pregnancy rates in endometriosis-related infertility: A retrospective matched cohort study	No control groups
Ashrafi	2018	The impact of the localisation of endometriosis lesions on ovarian reserve and assisted reproduction techniques outcomes	Surgical intervention
Park	2018	Could surgical management improve the IVF outcomes in infertile women with endometrioma?: a review	Surgical intervention
Alkudmani	2018	In Vitro Fertilization (IVF) Success Rates After Surgically Treated Endometriosis and Effect of Time Interval between Surgery and IVF	Surgical intervention
Mavrelos	2017	The impact of adenomyosis on the outcome of IVF–embryo transfer	No control group
Ferrero	2017	Impact of large ovarian endometriomas on the response to superovulation for in vitro fertilization: A retrospective study	No control group + not relevant outcomes
Guler	2017	The Impact of laparoscopic surgery of peritoneal endometriosis and endometrioma on the outcome of ICSI cycles	Surgical intervention
Leonardi	2016	Risk of miscarriage in women with endometriosis: insights from in vitro fertilization cycles	Not relevant outcomes
Motte	2016	In vitro fertilization outcomes after ablation of endometriomas using plasma energy: A retrospective case-control study	Surgical intervention
Fujii	2016	Assisted reproductive technology pregnancy complications are significantly associated with endometriosis severity before conception: a retrospective cohort study	Surgical intervention
Jacques	2016	Adverse pregnancy and neonatal outcomes after assisted reproductive treatment in patients with pelvic endometriosis: a case–control study	Surgical intervention
Benaglia	2016	Pregnancy outcome in women with endometriosis achieving pregnancy with IVF	Surgical intervention
Prasad	2015	Treatment options of endometriosis prior to in vitro fertilization/ intracytoplasmic sperm injection cycles to improve conception rate	Surgical and medical intervention
Park	2015	Effect of second-line surgery on in vitro fertilization outcome in infertile women with ovarian endometrioma recurrence after primary conservative surgery for moderate to severe endometriosis	Surgical intervention
Benaglia	2015	Intrafollicular iron and ferritin in women with ovarian endometriomas	Not relevant outcomes
Yan	2014	Effect of Adenomyosis on In Vitro Fertilization/Intracytoplasmic Sperm Injection Outcomes in Infertile Women: A Retrospective Cohort Study	Adenomyosis
Benaglia	2014	Asymptomatic adenomyosis and embryo implantation in IVF cycles	Adenomyosis
Pop Trajkovic	2014	*In vitro* fertilization outcome in women with endometriosis & previous ovarian surgery	Surgical intervention and medical treatment
Lee	2014	Surgical resection or aspiration with ethanol sclerotherapy of endometrioma before in vitro fertilization in infertilie women with endometrioma	Surgical intervention
Takebayashi	2013	Comparison of the outcome of *in vitro* fertilization after laparoscopic laser ablation surgery versus laparoscopic cystectomy for endometrioma	Surgical intervention
Dong	2013	The impact of endometriosis on IVF/ICSI outcomes	Surgical intervention

Takemura	2013	Increased risk of placenta previa is associated with endometriosis and tubal factor infertility in assisted reproductive technology pregnancy	Not relevant outcomes
Takashima	2013	Effects of bipolar electrocoagulation versus suture after laparoscopic excision of ovarian endometrioma on the ovarian reserve and outcome of in vitro fertilization	Surgical intervention
Stern	2013	Live birth rates and birth outcomes by diagnosis using linked cycles from the SART CORS database	Not relevant outcomes
Mekaru	2013	Effects of early endometriosis on IVF-ET outcomes	Surgical intervention
Guo	2012	Comparative study on the pregnancy outcomes of in vitro fertilization–embryo transfer between long-acting gonadotropin-releasing hormone agonist combined with transvaginal ultrasound-guided cyst aspiration and long-acting gonadotropin-releasing hormone agonist alone	Surgical intervention and medical treatment
Salim	2012	Adenomyosireduces pregnancy rates in infertile women undergoing IVF	Adenomyosis
Thalluri and Tremellen	2012	Ultrasound diagnosed adenomyosis has a negative impact on successful implantation following GnRH antagonist IVF treatment	Adenomyosis
Kuivasaari-Pirinen	2012	Adverse Outcomes of IVF/ICSI Pregnancies Vary Depending on Aetiology of Infertility	Not relevant outcomes
Kiran	2012	Does ovarian endometrioma affect the number of oocytes retrieved for in vitro fertilization?	Not relevant outcomes
Herzog	2012	The impact of endometriosis on in vitro fertilization outcome	No english language
Benaglia	2012	Pregnancy outcome in women with endometriomas achieving pregnancy through IVF	Surgical intervention
Ballester	2012	Cumulative pregnancy rate after ICSI– IVF in patients with colorectal endometriosis: results of a multicentre study	No control group
Opoien	2012	In vitro fertilization is a successful treatment in endometriosis-associated infertility	Surgical intervention
Martinez-Conejero	2011	Adenomyosis does not affect implantation, but is associated with miscarriage in patients undergoing oocyte donation	Oocyte donation
Scarselli	2011	Management of infertile women with endometriosis. What's the result of leaving ovarian endometrioma during IVF-Et cycles?	Surgical intervention
Youm	2011	In vitro fertilization and embryo transfer outcomes in relation to myometrial thickness	Adenomyosis
Costello	2011	The effect of adenomyosis on in vitro fertilisation and intra-cytoplasmic sperm injection treatment outcome	Adenomyosis
Almog	2011	Effects of ovarian endometrioma on the number of oocytes retrieved for in vitro fertilization	Not relevant outcomes
Dechanet	2011	Endometriosis and fertility: Results after surgery and Assisted Reproductive Technology (ART)	Surgical intervention
Reinblatt	2011	Effects of ovarian endometrioma on embryo quality	No data available
Bongioanni	2011	Ovarian endometriomas and IVF: a retrospective case-control study	No data available
Genovese	2011	Procreazione medicalmente assistita nelle pazienti affette da endometriosi: la nostra esperienza	Italian language
Mijatovic	2010	Adenomyosis has no adverse effects on IVF/ICSI outcomes in women with endometriosis treated with long-term pituitary down-regulation before IVF/ICSI	Medical treatment
Healy	2010	Surgical treatment of endometriosis: a prospective randomized double-blinded trial comparing excision and ablation	Surgical intervention
Barri	2010	Endometriosis-associated infertility: surgery and IVF, a comprehensive therapeutic approach	Surgical intervention

PRISMA 2020 Checklist

Section and Topic	Item #	Checklist item	Location where item is reported
**TITLE**			
Title	1	Identify the report as a systematic review.	1 title page
**ABSTRACT**			
Abstract	2	See the PRISMA 2020 for Abstracts checklist.	1
**INTRODUCTION**			
Rationale	3	Describe the rationale for the review in the context of existing knowledge.	**2**
Objectives	4	Provide an explicit statement of the objective(s) or question(s) the review addresses.	**2**
**METHODS**			
Eligibility criteria	5	Specify the inclusion and exclusion criteria for the review and how studies were grouped for the syntheses.	**2**
Information sources	6	Specify all databases, registers, websites, organisations, reference lists and other sources searched or consulted to identify studies. Specify the date when each source was last searched or consulted.	**3**
Search strategy	7	Present the full search strategies for all databases, registers and websites, including any filters and limits used.	**3**
Selection process	8	Specify the methods used to decide whether a study met the inclusion criteria of the review, including how many reviewers screened each record and each report retrieved, whether they worked independently, and if applicable, details of automation tools used in the process.	**3**
Data collection process	9	Specify the methods used to collect data from reports, including how many reviewers collected data from each report, whether they worked independently, any processes for obtaining or confirming data from study investigators, and if applicable, details of automation tools used in the process.	**3**
Data items	10a	List and define all outcomes for which data were sought. Specify whether all results that were compatible with each outcome domain in each study were sought (e.g. for all measures, time points, analyses), and if not, the methods used to decide which results to collect.	**3**
	10b	List and define all other variables for which data were sought (e.g. participant and intervention characteristics, funding sources). Describe any assumptions made about any missing or unclear information.	**3**
Study risk of bias assessment	11	Specify the methods used to assess risk of bias in the included studies, including details of the tool(s) used, how many reviewers assessed each study and whether they worked independently, and if applicable, details of automation tools used in the process.	**4**
Effect measures	12	Specify for each outcome the effect measure(s) (e.g. risk ratio, mean difference) used in the synthesis or presentation of results.	**4**
Synthesis methods	13a	Describe the processes used to decide which studies were eligible for each synthesis (e.g. tabulating the study intervention characteristics and comparing against the planned groups for each synthesis (item #5)).	**4**
	13b	Describe any methods required to prepare the data for presentation or synthesis, such as handling of missing summary statistics, or data conversions.	**4**
	13c	Describe any methods used to tabulate or visually display results of individual studies and syntheses.	**4**
	13d	Describe any methods used to synthesize results and provide a rationale for the choice(s). If meta-analysis was performed, describe the model(s), method(s) to identify the presence and extent of statistical heterogeneity, and software package(s) used.	**4**
	13e	Describe any methods used to explore possible causes of heterogeneity among study results (e.g. subgroup analysis, meta-regression).	**4**
	13f	Describe any sensitivity analyses conducted to assess robustness of the synthesized results.	**4**
Reporting bias assessment	14	Describe any methods used to assess risk of bias due to missing results in a synthesis (arising from reporting biases).	**4**
Certainty assessment	15	Describe any methods used to assess certainty (or confidence) in the body of evidence for an outcome.	**4**

PRISMA 2020 Checklist

Section and Topic	Item #	Checklist item	Location where item is reported
**RESULTS**			
Study selection	16a	Describe the results of the search and selection process, from the number of records identified in the search to the number of studies included in the review, ideally using a flow diagram.	5
	16b	Cite studies that might appear to meet the inclusion criteria, but which were excluded, and explain why they were excluded.	
Study characteristics	17	Cite each included study and present its characteristics.	5
Risk of bias in studies	18	Present assessments of risk of bias for each included study.	5
Results of individual studies	19	For all outcomes, present, for each study: (a) summary statistics for each group (where appropriate) and (b) an effect estimate and its precision (e.g. confidence/credible interval), ideally using structured tables or plots.	5
Results of syntheses	20a	For each synthesis, briefly summarise the characteristics and risk of bias among contributing studies.	5
	20b	Present results of all statistical syntheses conducted. If meta-analysis was done, present for each the summary estimate and its precision (e.g. confidence/credible interval) and measures of statistical heterogeneity. If comparing groups, describe the direction of the effect.	5
	20c	Present results of all investigations of possible causes of heterogeneity among study results.	5
	20d	Present results of all sensitivity analyses conducted to assess the robustness of the synthesized results.	6
Reporting biases	21	Present assessments of risk of bias due to missing results (arising from reporting biases) for each synthesis assessed.	6
Certainty of evidence	22	Present assessments of certainty (or confidence) in the body of evidence for each outcome assessed.	6
**DISCUSSION**			
Discussion	23a	Provide a general interpretation of the results in the context of other evidence.	6
	23b	Discuss any limitations of the evidence included in the review.	7
	23c	Discuss any limitations of the review processes used.	7
	23d	Discuss implications of the results for practice, policy, and future research.	7
**OTHER INFORMATION**			
Registration and protocol	24a	Provide registration information for the review, including register name and registration number, or state that the review was not registered.	Proispero CRD42023467215
	24b	Indicate where the review protocol can be accessed, or state that a protocol was not prepared.	not prepared
	24c	Describe and explain any amendments to information provided at registration or in the protocol.	na
Support	25	Describe sources of financial or non-financial support for the review, and the role of the funders or sponsors in the review.	none
Competing interests	26	Declare any competing interests of review authors.	none
Availability of data, code and other materials	27	Report which of the following are publicly available and where they can be found: template data collection forms; data extracted from included studies; data used for all analyses; analytic code; any other materials used in the review.	all on request

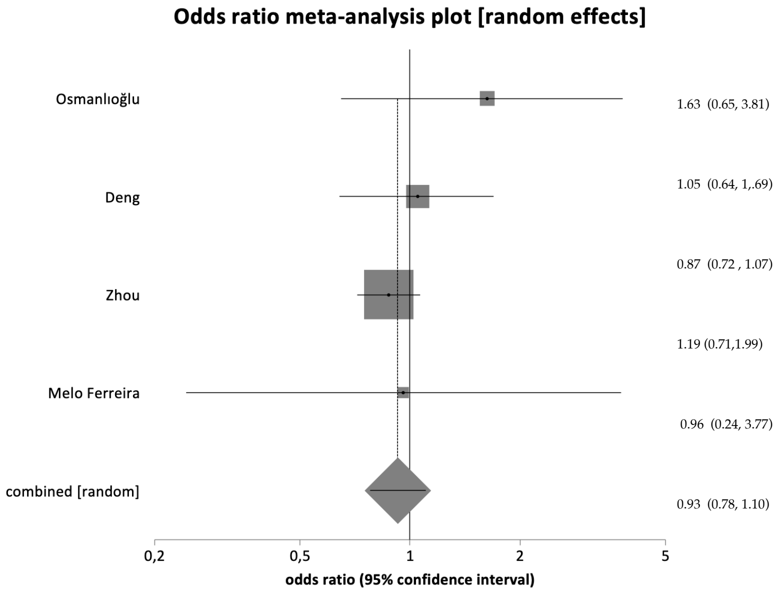

Supplementary Figure 1 Forest plot showing Live birth rate per transfer [23,24,25,30]

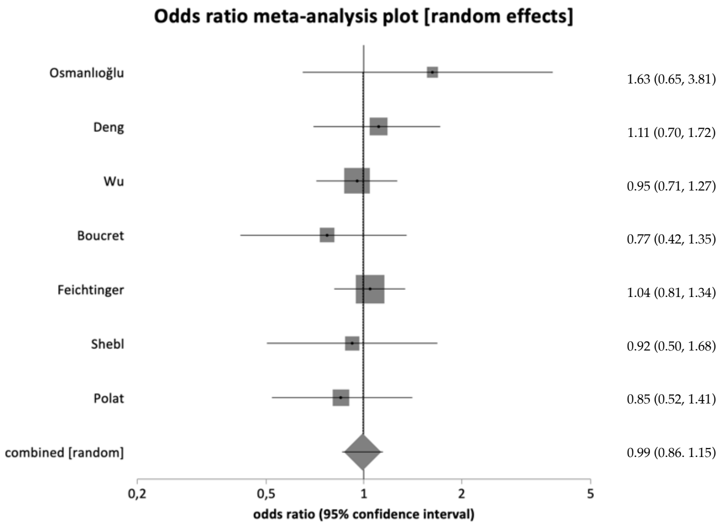

Supplementary Figure 2 Forest plot showing Live birth rate per cycle [23,24,26,27,29,35,44]

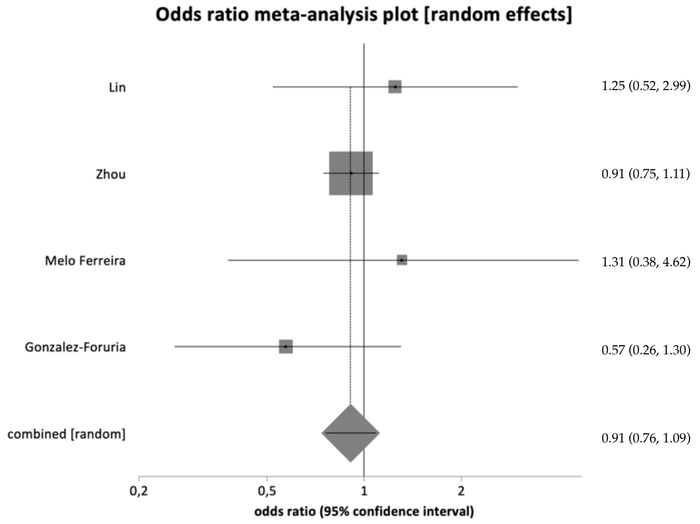

Supplementary figure 3 Forest plot showing Clinical pregnancy rate per transfer [25,30,36,50]

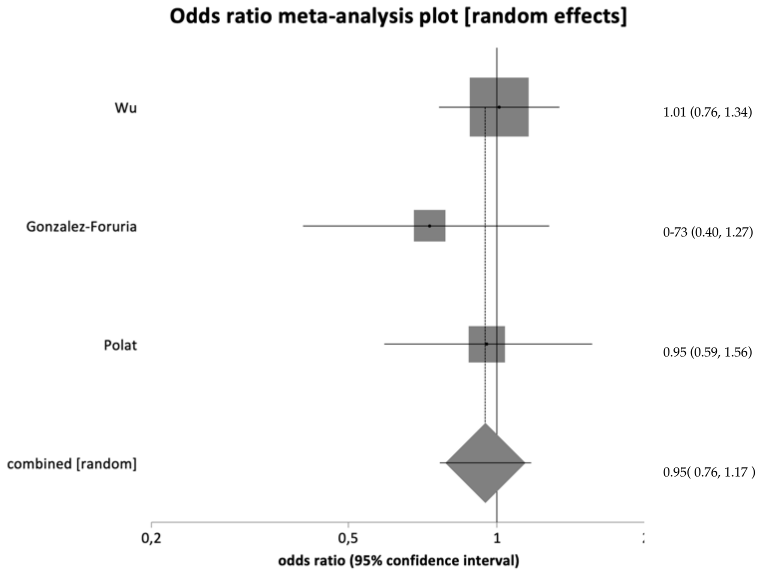

Supplementary figure 4 Forest plot showing Clinical pregnancy rate per cycle [26,36,44]-
